# Endocarp Morphology of *Premna* (Lamiaceae) in Thailand and Its Taxonomic Significance

**DOI:** 10.3390/plants14111706

**Published:** 2025-06-03

**Authors:** Jiratthi Satthaphorn, Alan J. Paton, Pornsawan Sutthinon, Charan Leeratiwong

**Affiliations:** 1 School of Science, Walailak University, Nakhon Si Thammarat 80160, Thailand; 2 Center of Excellence for Ecoinformatics, Walailak University, Nakhon Si Thammarat 80160, Thailand; 3 Science Directorate, Royal Botanic Gardens, Kew, Richmond TW9 3AE, UK; 4 Department of Botany, Faculty of Science, Kasetsart University, Bangkok 10900, Thailand; 5 Division of Biological Science, Faculty of Science, Prince of Songkla University, Songkhla 90110, Thailand

**Keywords:** drupe, micromorphology, pericarp, Premnoideae, sculpturing, taxonomy

## Abstract

Fruits and endocarps of 21 species within the genus *Premna* (Lamiaceae) in Thailand were examined using light (LM) and scanning electron microscopy (SEM) to evaluate taxonomic relevance. Overall, mature fruits were classified into two types: fully developed mericarp (fruit type I) and partly developed mericarp (fruit type II), with three shape patterns: broadly obovoid, narrowly obovoid, and clavoid. Fruit size ranged from 1.52 to 7.48 mm in length and 0.98 to 7.71 mm in width. In LM investigations, the endocarps were classified into three types based on the presence and shape of the protruding structure: saccate-like (protrusion type I), thorn-like (protrusion type II), and no protrusion (protrusion type III). The examination of endocarps under SEM showed that they consist of multilayers of sclerenchyma cells. The shape of the sculpturing cells on the endocarp surface can be divided into two patterns: irregular tetragonal and polygonal, with distinct or obscure straight cell faces. The morphological comparison and phenetic analyses using factor analysis of mixed data (FAMD) show that fruit and endocarp characteristics of *Premna* hold significant taxonomic value for distinguishing certain related species and classifying within the genus in Thailand. From the first two FAMD dimensions, fruit shape, shape of sculptured cells on the endocarp, and protrusion type of the endocarp are considered as the most significant contributing variables. The findings also support the reinstatement of species previously synonymized with *P. serratifolia*, namely *P. cordifolia*, *P. paniculata*, and *P. punctulata*.

## 1. Introduction

*Premna* L. is a large genus of the subfamily Premnoideae within the family Lamiaceae, comprising approximately 130 species found across tropical and subtropical regions of Asia, Africa, and Australia [1,2,3,4]. The genus was first described by Linnaeus [5] based on the widely distributed species, *P. serratifolia* L. The genus and several genera, e.g., *Callicarpa* L., *Clerodendrum* L., *Glossocarya* Wall. ex Griff., *Tectona* L.f., and *Vitex* L., were initially classified under the family Verbenaceae, but they were later transferred to the family Lamiaceae [2,6]. Phylogenetic relationships based on molecular evidence showed that representatives of *Premna* form a monophyletic clade, placed within the subfamily Premnoideae with its related genera, *Gmelina* L. and *Cornutia* L. [3,4,7].

The genus is distinct within the family Lamiaceae by having simple, opposite leaves, terminal inflorescences, zygomorphic to sub-actinomorphic flowers, a two-lipped calyx with four to five lobes or an entire, short, and funnel-shaped corolla, typically with four lobes (rarely five lobes), dense white hairs on the inner corolla surface, and drupaceous fruit [8,9,10]. However, morphological distinctions between species are subtle and challenging to identify, as they share many similar characteristics. Early taxonomic studies of *Premna* by Fletcher [11], Chen and Gilbert [12], Leeratiwong et al. [8]**,** and de Kok [9,10] primarily relied on the floral parts for the identification of species, which are usually small and fragile. These characteristics are sometimes inadequate for accurate identification and for interpreting the phylogenetic relationships within the genus [13,14]. Consequently, new diagnostic features are required to improve the species circumscription and classification at the interspecies level.

Fruit characteristics are crucial for taxonomic identification and classification within the family Lamiaceae [15]. Several taxonomic studies have typically utilized fruiting features to delimit species within genera as one of the important morphological characters, such as *Callicarpa*, *Glossocarya*, *Petraeovitex* Oliv., and *Teijsmanniodendron* Koord. [8,10,16,17]. Additionally, several studies have focused on detailed fruit characteristics across various tribes [18,19,20,21,22,23] and different genera, such as *Dracocephalum* L., *Lamium* L., *Monarda* L., *Ocimum* L., *Orthosiphon* Benth., *Phlomoides* Moench, *Plectranthus* L′Hér., *Salvia* L., *Satureja* L., *Stachys* L., and *Vitex* L. [14,24,25,26,27,28,29,30,31,32,33,34,35,36,37]. The results from these studies suggested that taxonomic evaluation can benefit from examining fruit morphology and micromorphology. However, most studies have focused on taxa that produce dry fruits, and no studies have yet examined detailed fruit morphology as a significant taxonomic feature within *Premna*.

In Thailand, Leeratiwong et al. [8] recognized 22 species of *Premna*. This study employed the shapes of fruits to group species in an early identification key couplet. This morphological approach aligns with the classification methods used for Malesian species [9,10]. We initially noticed that the shapes and sizes of fruits are useful to distinguish, at the species level, *Premna* in Thailand. Moreover, the external surface of fresh fruits appeared smooth for all species at first glance, but the dried fruits revealed a rough texture caused by the underlying shape of the endocarp becoming prominent and different across species. These observations obtained from the morphological study of mature fruits, as well as morphological and micromorphological investigations of endocarps, are hypothesized to support species delimitation within *Premna* in Thailand. Phenetic analyses were also carried out in this study using a combination of qualitative and quantitative morphological characters, as earlier studies have shown to be useful for differentiating closely related species [38,39,40].

This study aims to (1) provide information on the shape and size of mature fruits of *Premna* in Thailand; (2) illustrate details of endocarp morphology and micromorphology of *Premna* in Thailand using light and scanning electron microscopy; and (3) elucidate the taxonomic significance and relationships within *Premna* based on available fruit characteristics.

## 2. Results

**Figure 1:**
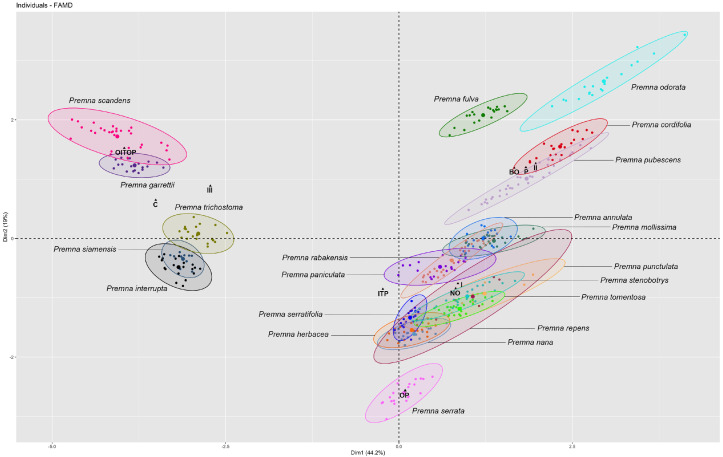
Two-dimensional scatter plot depicting the coordinates of each individual from 423 accessions of *Premna* in Thailand in the FAMD analyses. Each dot represents one individual accession. The abbreviations of qualitative characters indicated in bold font: shape (C = clavoid, BO = broadly obovoid, NO = narrowly obovoid); shape of sculptured cells of endocarp (ITP = irregular tetragonal and polygonal, OITOP = obscurely irregular tetragonal and obscurely polygonal, OP = obscurely polygonal, P = polygonal); and protrusion type of endocarp (I, II, III).

**Figure 2:**
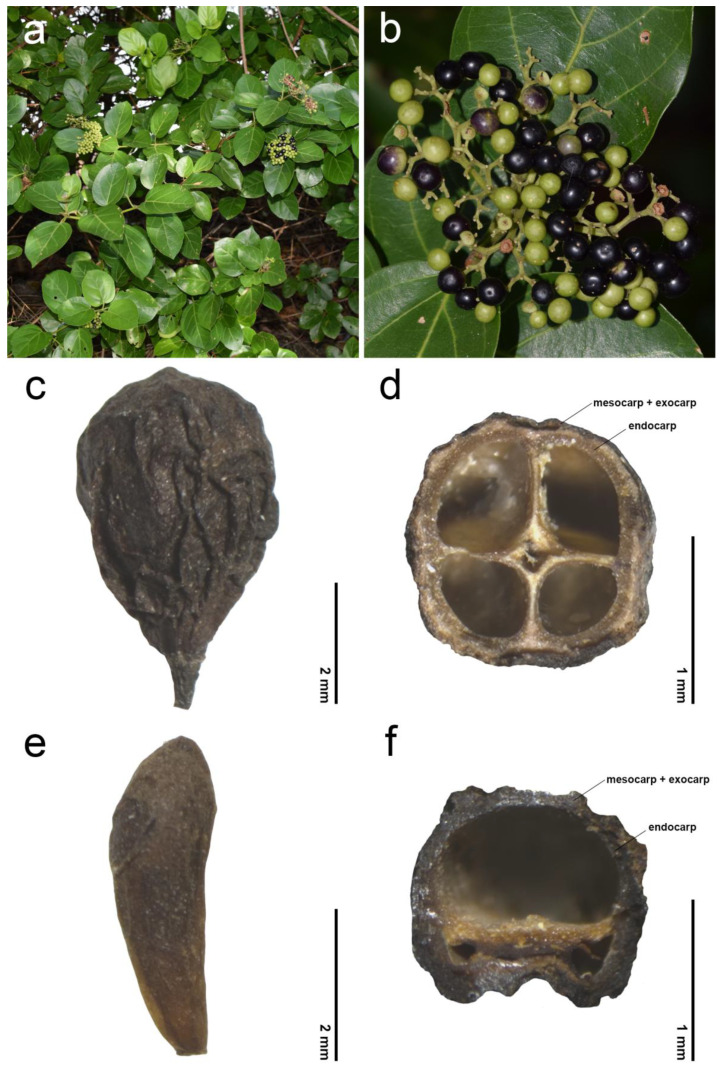
General fruit morphology of *Premna* in Thailand. (**a**) Fruiting branch of *P. serratifolia*; (**b**) infructescence of *P. serratifolia*; (**c**) mature fruit type I (*P. serratifolia*) (LM); (**d**) cross-section of mature fruit type I (*P. serratifolia*) (LM); (**e**) mature fruit type II (*P. scandens*) (LM); (**f**) cross-section of mature fruit type II (*P. scandens*) (LM).

**Figure 3:**
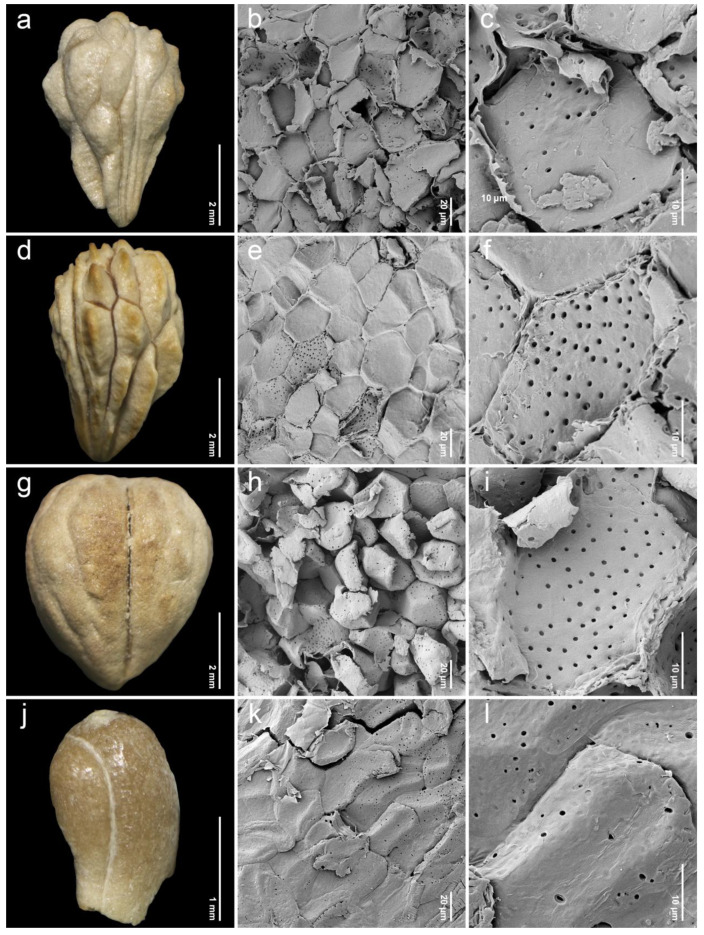
Endocarp micromorphology of *Premna* in Thailand. (**a**,**d**,**g**,**j**) Endocarp outline (LM); (**b**,**e**,**h**,**k**) endocarp surface (SEM); (**c**,**f**,**i**,**l**) closed-up pitted cells on endocarp surface (SEM). (**a**–**c**) *P. annulata*; (**d**–**f**) *P. cordifolia*; (**g**–**i**) *P. fulva*; (**j**–**l**) *P. garrettii*.

**Figure 4:**
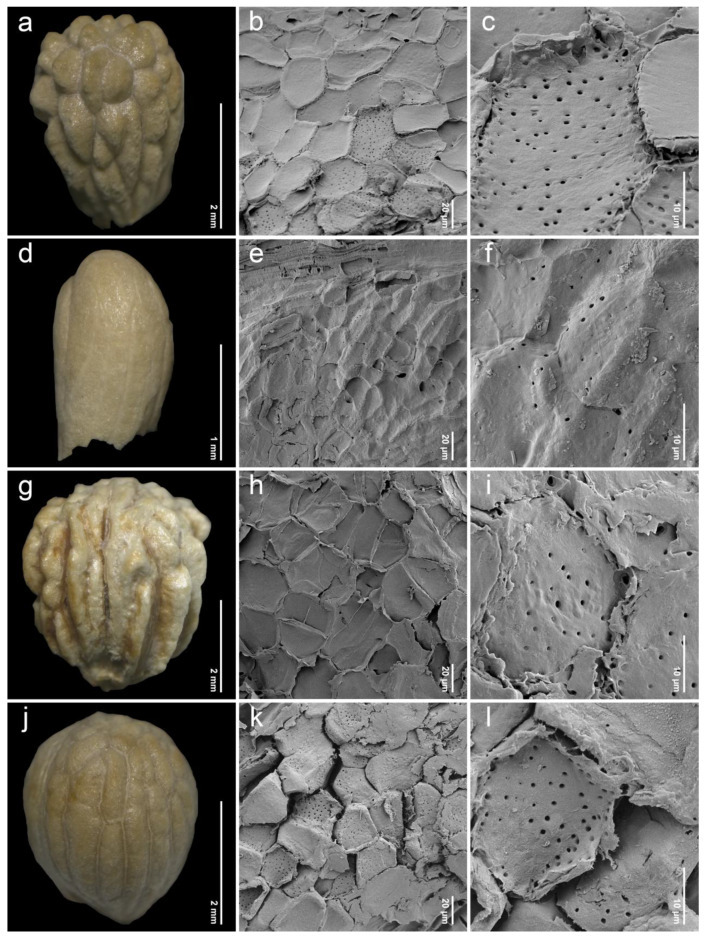
Endocarp micromorphology of *Premna* in Thailand. (**a**,**d**,**g**,**j**) Endocarp outline (LM); (**b**,**e**,**h**,**k**) endocarp surface (SEM); (**c**,**f**,**i**,**l**) closed-up pitted cells on endocarp surface (SEM). (**a**–**c**) *P. herbacea*; (**d**–**f**) *P. interrupta*; (**g**–**i**) *P. mollissima*; (**j**–**l**) *P. nana*.

**Figure 5:**
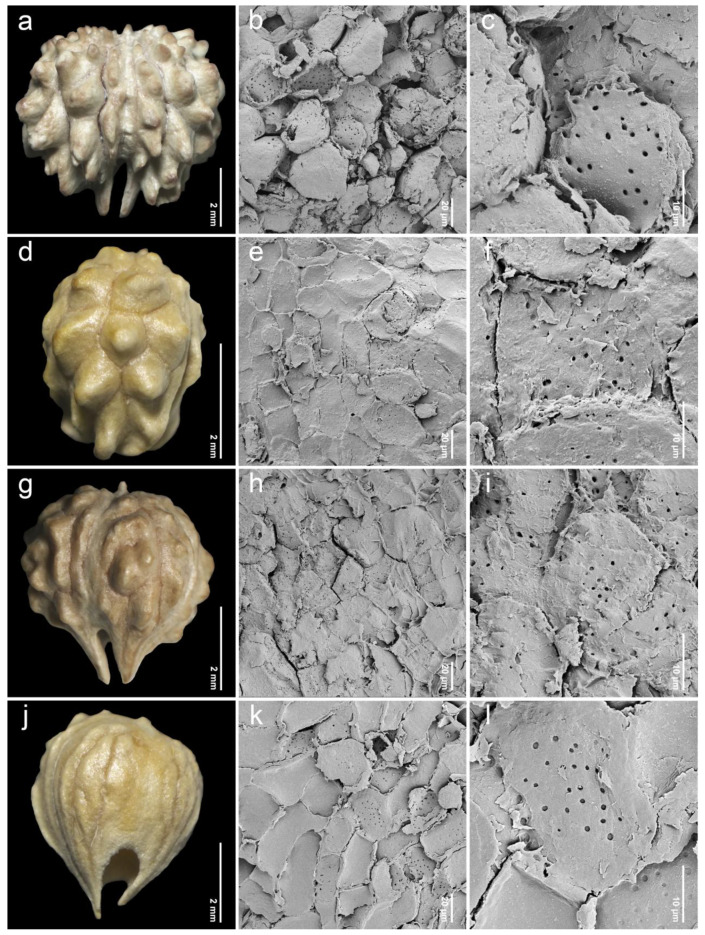
Endocarp micromorphology of *Premna* in Thailand. (**a**,**d**,**g**,**j**) Endocarp outline (LM); (**b**,**e**,**h**,**k**) endocarp surface (SEM); (**c**,**f**,**i**,**l**) closed-up pitted cells on endocarp surface (SEM). (**a**–**c**) *P. odorata*; (**d**–**f**) *P. paniculata*; (**g**–**i**) *P. pubescens*; (**j**–**l**) *P. punctulata*.

**Figure 6:**
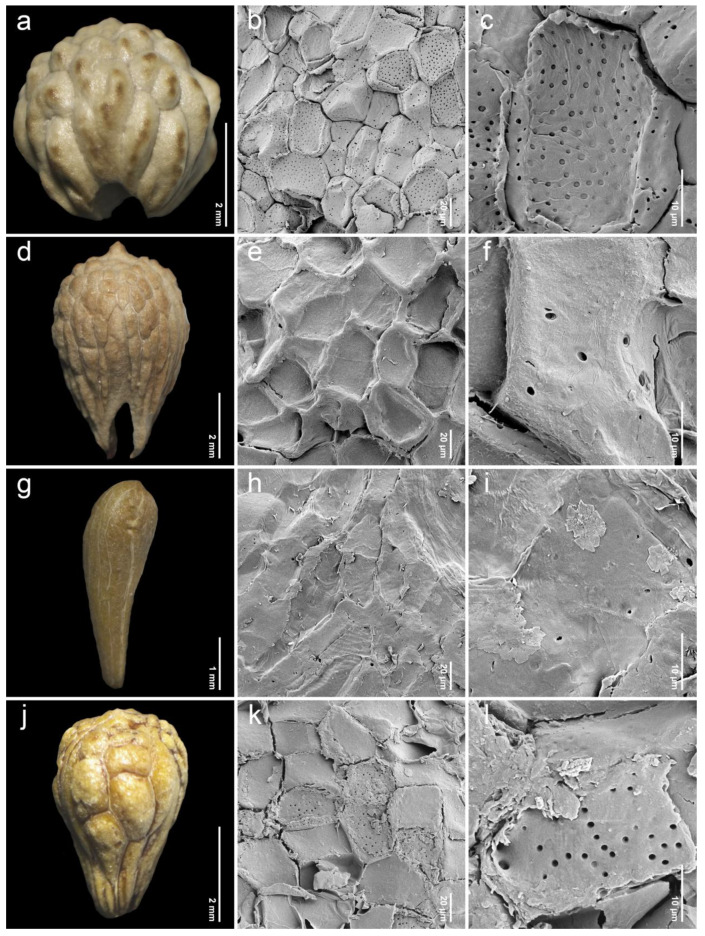
Endocarp micromorphology of *Premna* in Thailand. (**a**,**d**,**g**,**j**) Endocarp outline (LM); (**b**,**e**,**h**,**k**) endocarp surface (SEM); (**c**,**f**,**i**,**l**) closed-up pitted cells on endocarp surface (SEM). (**a**–**c**) *P. rabakensis*; (**d**–**f**) *P. repens*; (**g**–**i**) *P. scandens*; (**j**–**l**) *P. serrata*.

**Figure 7:**
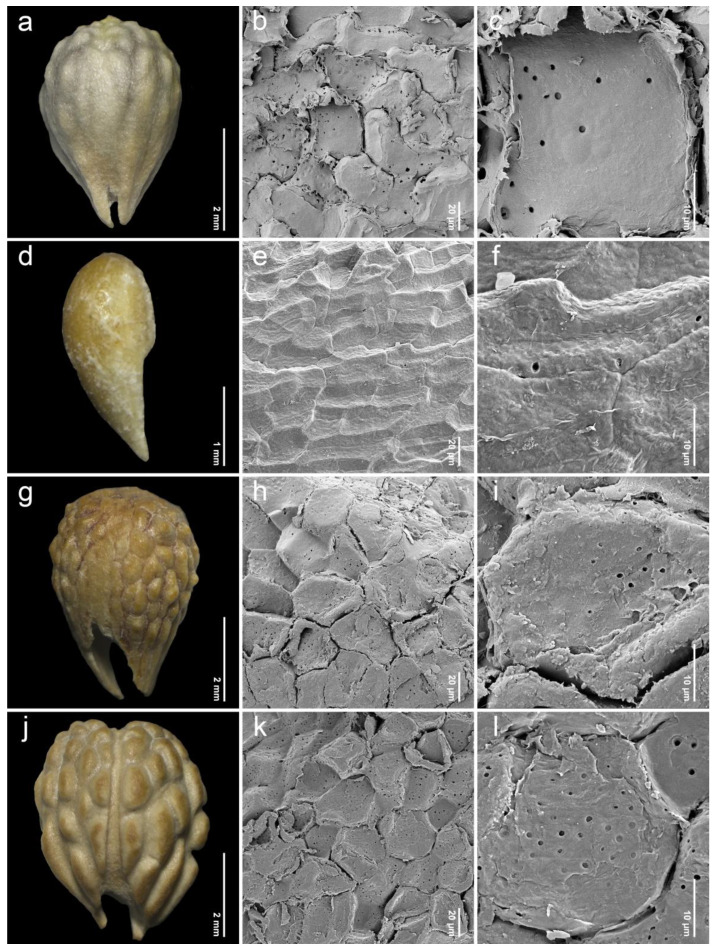
Endocarp micromorphology of *Premna* in Thailand. (**a**,**d**,**g**,**j**) Endocarp outline (LM); (**b**,**e**,**h**,**k**) endocarp surface (SEM); (**c**,**f**,**i**,**l**) closed-up pitted cells on endocarp surface (SEM). (**a**–**c**) *P. serratifolia*; (**d**–**f**) *P. siamensis*; (**g**–**i**) *P. stenobotrys*; (**j**–**l**) *P. tomentosa*.

**Figure 8:**
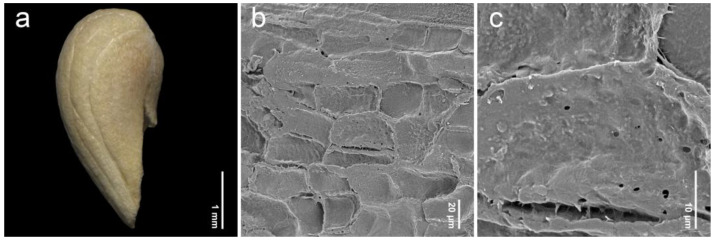
Endocarp micromorphology of *Premna* in Thailand. (**a**) Endocarp outline (LM); (**b**) endocarp surface (SEM); (**c**) closed-up pitted cells on endocarp surface (SEM). (**a**–**c**) *P. trichostoma*.

**Figure 9:**
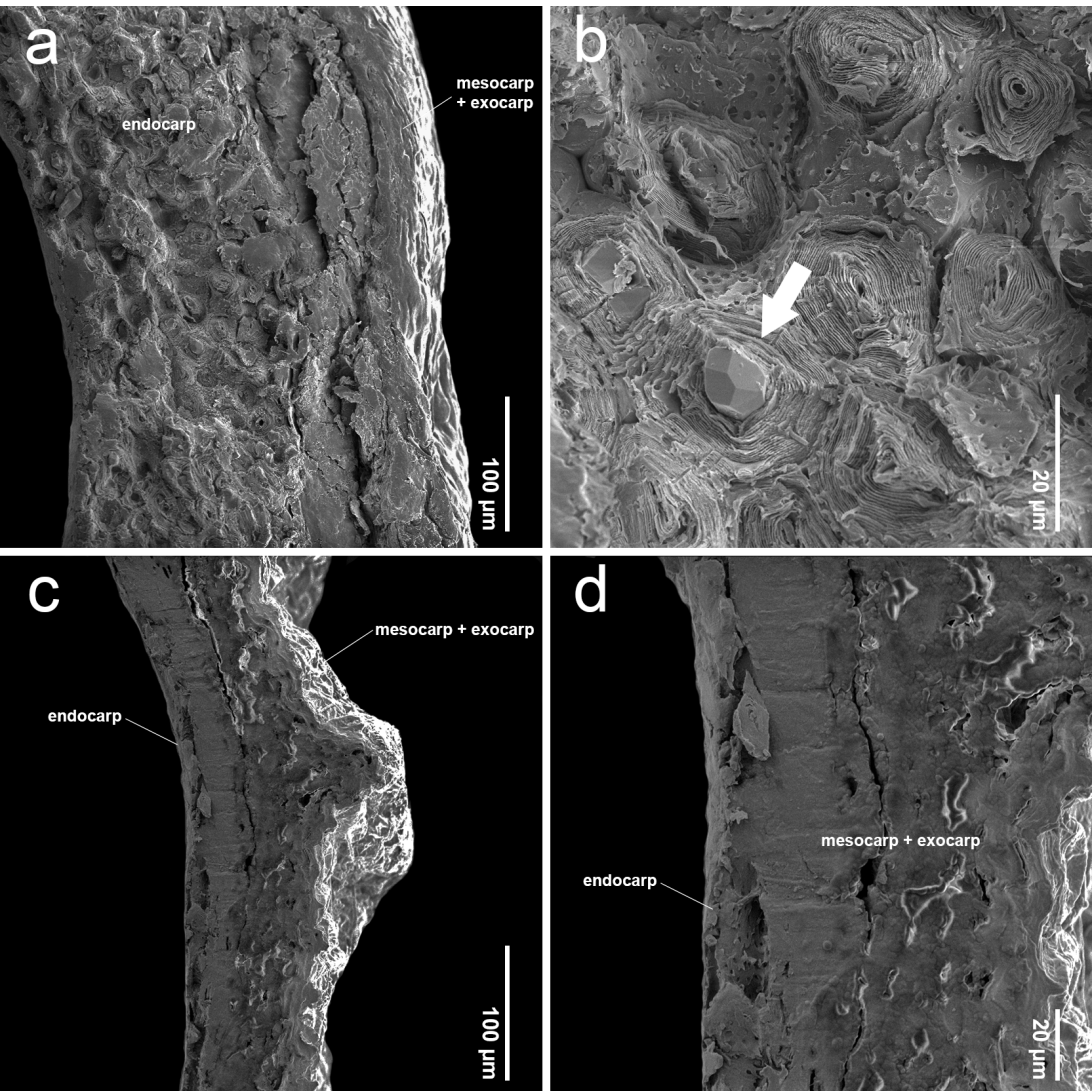
SEM micrographs showing cross-sections of *P. serratifolia* and *P. scandens* mature fruits. (**a**) Pericarp layer of *P. serratifolia* fruit; (**b**) lignified cell walls and prismatic crystals (arrow) in endocarp layer of *P. serratifolia*; (**c**) pericarp layer of *P. scandens*; (**d**) closed-up pericarp layer of *P. scandens*.

The morphological investigations and phenetic analyses of the fruits and endocarps of *Premna* in Thailand are summarized in Table 1 and illustrated in Figure 1, Figure 2, Figure 3, Figure 4, Figure 5, Figure 6, Figure 7, Figure 8 and Figure 9. The mature fruits were identified as a drupe, consisting of three pericarp layers: the smooth and fleshy outer layer (exocarp), the viscous and fibrous middle layer (mesocarp), and the dry woody innermost layer (endocarp). The fleshy exocarp was usually fused with the mesocarp and woody endocarp, covering four locules, each accommodating a single membranous seed (Figure 2). *Premna garrettii* H.R.Fletcher, *P. interrupta* Wall. ex Schauer, *P. scandens* Roxb., *P. siamensis* H.R.Fletcher, and *P. trichostoma* Miq. are exceptions. These species exhibited unequal sizes of locules, and only one developed a seed found in the largest locule (Figure 2f).

### 2.1. General Information of Fruit Morphology

The exocarp of *Premna* in Thailand was consistently light green when young, turning black upon maturity. In some cases, the exocarp of *P. serratifolia* appeared dark purple (Figure 2a,b). In this study, mature fruits were divided into two types based on the development of each mericarp. Type I referred to fruits exhibiting fully developed mericarps and four equal-sized locules (Figure 2c,d). Within Type I, the fruit shape can be categorized into two patterns based on the outline and average ratio between the length (L) and width (W): (1) broadly obovoid (L/W ratio up to 1.10) and (2) narrowly obovoid (L/W ratio between 1.11 and 1.50). Type II was represented by fruits with unequally developed mericarps, resulting in one large locule and three smaller ones (Figure 2e,f). The shape of fruit type II was always clavoid (narrowly club-shaped, L/W ratio more than 1.50). Overall, the lengths of fruits ranged from 1.52 to 2.49 (2.11 ± 0.27 average) mm long in *P. interrupta* to 4.94–7.48 (5.79 ± 0.72 average) mm long in *P. odorata* Blanco (Table 1). The width ranged from 0.98 to 1.37 (1.13 ± 0.12 average) mm wide in *P. interrupta* to 4.65–7.71 (5.90 ± 0.72 average) mm wide in *P. odorata* (Table 1). The length/width (L/W) ratio was variable from 0.88 to 1.17 (0.98 ± 0.07 average) mm in *P. odorata* to 1.90–3.81 (2.94 ± 0.48 average) mm in *P. scandens* (Table 1).

### 2.2. Endocarp Morphology and Micromorphology

After removing the exocarp and mesocarp layers, the woody endocarp exposed a protruding structure that can be classified into three patterns based on LM (stereomicroscope) observations. Protrusion type I referred to the endocarp featuring saccate-like (rounded and swollen) protrusions, which were observed in 11 species, particularly at the distal end of the endocarp, such as *P. annulata* H.R.Fletcher (Figure 3a), *P. herbacea* Roxb. (Figure 4a), *P. nana* Collett & Hemsl. (Figure 4j), *P. pubescens* Blume (Figure 5g), *P. serrata* H.R.Fletcher (Figure 6j), and *P. serratifolia* (Figure 7a). Protrusion type II indicated the endocarp with thorn-like protrusions (sharpened and tapered, sometimes forming vertical ridges), found in three species, namely *P. cordifolia* Roxb. (Figure 3d), *P. odorata* (Figure 5a), and *P. paniculata* H.R.Fletcher (Figure 5d). Protrusion type III was characterized by the absence of protruding structures of the endocarp, found in six species, including *P. fulva* Craib (Figure 3g), *P. garrettii* (Figure 3j), *P. interrupta* (Figure 4d), *P. scandens* (Figure 6g), *P. siamensis* (Figure 7d), and *P. trichostoma* (Figure 8a). All these species having protrusion type III correspond with clavoid fruits (fruit type II), except for *P. fulva,* which belongs to fruit type I.

The SEM images revealed characteristics of sculptured cells on the endocarp surface and in the organization within endocarp tissue. The sculptured cells on the endocarp surface are generally polygonal or a combination of polygonal and irregular tetragonal shapes, with straight faces or occasionally indistinct faces (Table 1, Figure 3, Figure 4, Figure 5, Figure 6, Figure 7 and Figure 8). Most species exhibited distinct cell boundaries, whereas the cells of *P. garrettii*, *P. scandens*, and *P. serrata* had indistinct faces ( Figure 3l, Figure 6i, and Figure 6l, respectively). All species exhibited pitted cell walls preserved across most regions of the surface. However, the endocarps of a clavoid fruit (fruit type II) showed less-pitted cell walls, compared to those within the fruit type I. In the cross-sections of fruit specimens, the endocarp of fruit type I was approximately 150–250 µm thick, consisting of several layers of sclerenchyma cells and crystalized (prismatic) cells (Figure 9a,b). In contrast, the endocarp of fruit type II was about 10–90 µm thick, with fewer layers of sclerenchyma cells (Figure 9c,d). In general, the sclerenchyma cells were composed of several layers of secondary cell walls deposited on the inner side of the primary wall, reducing the cell cavity.

### 2.3. Phenetic Analyses

The first two dimensions of factor analysis of mixed data (FAMD) explained 44.2% (Dim1) and 19% (Dim2), accounting for 63.2% of the overall information (Figure 1). The results of the FAMD, showing the distribution of 423 accessions (each dot represents one individual accession), were plotted on a two-dimensional scatter plot. All representatives from 21 species of *Premna* were shown with different colors and labels. The variables of length, width, and length-to-width ratio were quantitative characters, while fruit shape, shape of the sculptured cells on the endocarp, and protrusion type of the endocarp were qualitative characters. Among these six characters associated with the first two dimensions, the shape of the sculptured cells on the endocarp, fruit shape, and the protrusion type of the endocarp were identified as the most significant contributing variables. In contrast, the contributions of width, length-to-width ratio, and length gradually declined, respectively (Figure S1). The distribution of the *Premna* accessions, characterized by fruit type I, including *P. fulva, P. odorata*, and *P. serrata*, was separately plotted. However, certain accessions exhibited overlapping groupings, such as *P. annulata*, *P. mollissima* Roth, *P. rabakensis* Moldenke, and *P. paniculata*; *P. herbacea*, *P. nana*, *P. punctulata* C.B.Clarke, *P. repens* H.R.Fletcher, *P. serratifolia*, *P. stenobotrys* Merr., and *P. tomentosa* Willd. (Figure 1). Accessions of *Premna* having fruit type II were separated into two loose clusters: one comprising *P. garrettii* and *P. scandens*, and the other consisting of *P. interrupta*, *P. siamensis*, and *P. trichostoma*. These two clusters were clearly isolated from the remaining species, producing fruit type I.

## 3. Discussion

### 3.1. Fruit Diversity of the Genus Premna in Thailand 

Several studies have demonstrated that fruit characteristics are important taxonomic features for species identification within the family Lamiaceae [14,15,21,41]. Our study provides the first details of fruit and endocarp characteristics of *Premna* in Thailand and contributes information for further systematic study (Table 1, Figure 1, Figure 2, Figure 3, Figure 4, Figure 5, Figure 6, Figure 7, Figure 8 and Figure 9). The investigation of the fleshy drupe provides new diagnostic characters for identification and classification within the genus *Premna*, especially type, shape, and size, as preliminarily supported by Leeratiwong et al. [8] and de Kok [9,10]. Three shapes of *Premna* fruits in Thailand, based on the average length and width (broadly obovoid, narrowly obovoid, and clavoid), particularly align with the species descriptions provided by previous studies [8,9,10]. However, Leeratiwong et al. [8] classified fruit shapes into two patterns in an early couplet of an identification key: (1) ovoid, obovoid, and subglobose shapes and (2) ellipsoid and obovoid–ellipsoid shapes. De Kok [9] identified two main fruit shapes for Malesian species: globose and clavoid. Although both studies classified fruit shapes into two main groups, the recognition of these shapes was different. For example, the fruit shape of *P. trichostoma* was described by Leeratiwong et al. [8] as ellipsoid to obovoid–ellipsoid, while de Kok [9] treated it as clavoid. To avoid subjectivity in shape appreciation we suggest the use of average length-to-width ratios together with the external observation to homogenize the recognition of fruit shapes of *Premna* in Thailand: (1) broadly obovoid (L/W ratio up to 1.10), (2) narrowly obovoid (L/W ratio between 1.11 and 1.50), and (3) clavoid (L/W ratio greater than 1.50).

Fruits with fully developed thick mericarps and four equal locules (fruit type I) are most common in Thai species: 12 species have a narrowly obovoid shape, and four species exhibit a broadly obovoid shape (Figure 2c,d). Fruits with partially developed thin mericarps and unequal locules (fruit type II) occur in five species, producing a clavoid shape (Figure 2e,f). This information is useful to classify species, along with three patterns of fruit shapes. The utility of fruit shapes for the significant grouping species has been reported in some genera within the family Lamiaceae, such as the genera *Elsholtzia* Willd. and *Salvia*. On the other hand, fruit shapes display no significant differences in some genera, such as *Collinsonia* L., *Keiskea* Miq., *Lycopus* L., *Mosla* (Benth.) Buch.-Ham. ex Maxim, and *Perilla* L. [21,41,42].

The size of the fruits serves as a distinguishing characteristic among different species of *Premna* in Thailand (Figure 1). Most species exhibited an average fruit length between 3 mm to 6 mm, whereas only two species, *P. interrupta* and *P. siamensis*, had a shorter average length of 2 mm to 3 mm long (Table 1). The fruit width of *P. garrettii*, *P. interrupta*, *P. scandens*, *P. siamensis*, and *P. trichostoma* ranged between 1 mm and 2 mm, while most other species had an average width between 2 mm and 6 mm wide (Table 1). The narrower fruit size of these five species corresponds with the clavoid shape of the fruit type II, allowing these species to be morphologically delimited from other species (see taxonomic significance below). The difference in fruit size to distinguish species is supported by the findings of Jeon et al. [21], which identifies species belonging to the genus *Elsholtzia*.

After soaking the mature fruits of *Premna*, removing the mesocarp and exocarp revealed no myxocarpic (mucilage-producing) properties. This mucilage typically aids in anchoring fruits to the soil in dry habitats [2,43,44,45]. Previous studies have documented this property in representatives of the tribes Elsholtzieae and Mentheae within the subfamily Nepetoideae, which produce mucilage from dry mericarps [21,35,41,42,46]. In *Premna*, both fruit types I and II are potentially at least associated with animals or birds due to their fleshy pericarp layers. However, the presence of protruding structures from the endocarp, particularly in fruit type I, may facilitate substrate anchoring and influence seed dispersal modes, as suggested by Paton [47].

### 3.2. Comparative Study of Endocarp Morphology and Micromorphology 

The examinations of endocarp characteristics to study morphology and micromorphology using LM and SEM illustrated new diagnostic features among *Premna* in Thailand (Figure 2, Figure 3, Figure 4, Figure 5, Figure 6, Figure 7 and Figure 8) that have not been previously described. As the endocarp is enclosed in two fused fleshy mesocarp and exocarp, this characteristic is likely less influenced by environmental factors [48]. Three distinct types of endocarp protrusions, namely saccate-like (protrusion type I), thorn-like (protrusion type II), and no protrusion (protrusion type III), can be used to classify species. All species in Thailand with fruit type I equally develop four-seeded fruits within protruding endocarps, except *P. fulva,* which lacks endocarp protrusions. The presence of endocarp protrusions plays several important roles in anchoring the substrates, including the prevention of fruit herbivores or seed destruction caused by animals, insects, and other unstable environmental factors [42,47,49]. This strategy may enhance seed viability and increase the chances of germination from seeds dispersed by animals. The case of clavoid fruits (fruit type II), which corresponded to non-protruding endocarps (protrusion type III), shows that all species that possess clavoid fruits with a thin fleshy layer and a single functional seed. In our study, these fruits display less attractive characteristics, including a reduction in size and fleshiness, making them less associated with animals [50,51]. This strategy may serve the protection of single-seeded fruits from frugivores (Figure 2e,f).

Investigations under SEM revealed the characteristics of sculptured cells on the endocarp surface and the components within the tissue layers (Figure 3, Figure 4, Figure 5, Figure 6, Figure 7 and Figure 8). The surface displays sculptured cells made up of uniform or irregular shapes, primarily tetragonal to polygonal, with straight or occasionally indistinct faces. In all species, the cell surfaces are generally smooth and have pitted cell walls, indicating a connection to the mesocarp cells and seeds [52]. This porous structure enables the transport of air, water, or other nanofluids through the plant structure, supporting imbibition and cellular respiration during embryo germination [52,53,54,55,56]. The endocarp surface associated with fruit type I is likely to more frequently exhibit pitted cell walls, which correlate with the shape and thickness of the pericarps. Fruit type II and the lack of endocarp protrusion (protrusion type III) show fewer pits. However, further anatomical studies are needed to investigate the functional role of the pits on the endocarp surface.

The SEM images of the pericarp cross-section revealed several layers of sclerenchyma cells, which are found exclusively in the endocarp of *Premna* and are typically observed in the fruiting structure in various plants [29,37,56,57,58,59]. These sclerenchyma cells are characterized by multiple layers of secondary cell walls, formed within the primary cell wall. The presence of secondary cell walls results in the absence of a protoplast due to the reduced cell cavity (Figure 9a,b). The secondary cell walls are composed of aggregated cellulose microfibrils arranged in multiple layers, which develop at different stages of cell differentiation [60,61]. The functions of these sclerenchyma cells are to promote the mechanical stability and reduce the permeability of the fruit wall due to the lignified contents [62,63]. Our study found that fruit type I exhibits more layers of sclerenchyma cells compared to fruit type II, enhancing seed protection and resilience in unstable environments from the natural habitats, such as exposure to sunlight or salt spray from the sea (e.g., *P. annulata*, *P. cordifolia*, *P. paniculata*, *P. punctulata*, *P. repens*, and *P. serratifolia*). The presence of prismatic crystals in the endocarp of fruit type I (Figure 9b) suggests a role for the plant related to defense against herbivores and chewing insects, as well as in metal and calcium metabolism and homeostasis [58,64,65,66,67,68]. We observed that species producing clavoid fruits (fruit type II) were primarily found in mesic evergreen forests, near streams or waterfalls [8], where conditions are presumably favorable for seed survival and germination. This seems to reduce the need for protection in fruit type II compared to fruit type I, resulting in fewer sclerenchyma layers and a lower number of fertile seeds.

### 3.3. Taxonomic Significance

Although most *Premna* in Thailand have not yet been included in phylogenetic studies, we discovered that fruit shapes hold limited taxonomic significance across species. The first two FAMD dimensions accounted for 63.2% of the total information (Figure 1). The analyses successfully separated several taxa based on the overall data, thereby confirming the usefulness of FAMD in detecting taxonomic patterns associated with fruit characteristics. While a minimum of 20 specimens was initially targeted for each taxon, three taxa, namely *P. punctulata*, *P. repens*, and *P. siamensis*, were represented by 6 to 10 specimens due to limited availability. However, FAMD analyses revealed considerable variability within these taxa.

For species with fruit type I, fruit characters proved useful for distinguishing *P. fulva*, *P. odorata*, and *P. serrata* at the species level, based on differences in fruit shape, size, shape of sculptured cells on the endocarp, and protrusion type of the endocarp. These findings corresponded with the clearly separated FAMD accession groupings shown in Figure 1. On the contrary, some species bearing fruit type I with narrowly and broadly obovoid fruits lacked distinctive fruit morphological characters to separate species [8], consistent with the overlapping distribution plots of their accessions (e.g., *P. annulata*, *P. mollissima*, *P. paniculata*, *P. rabakensis*; *P. cordifolia* and *P. pubescens*; *P. herbacea*, *P. nana*, *P. punctulata*, *P. repens*, *P. serratifolia*, *P. stenobotrys*, *P. tomentosa*). The species having a clavoid fruit shape, referring to fruit type II, namely *P. garrettii*, *P. interrupta*, *P. scandens*, *P. siamensis*, and *P. trichostoma,* corresponded to having unequally developed mericarps (Figure 2e,f). In addition, these species share some morphological features, such as short calyx lobes less than 1 mm long and white to greenish-white or creamy-white corollas [8]. The notable distinctions in fruit shape and size among these species were consistent with the differentiation revealed by FAMD analyses when compared to other *Premna* in Thailand (Figure 1). This clavoid fruit can be observed in many species from neighboring regions, such as *P. bracteata* Wall. ex C.B.Clarke, *P. oblongata* Miq., *P. parasitica* Blume, and *P. regularis* H.J.Lam [9,12].

Fruit size can be utilized as a diagnostic character for some morphologically related species in Thailand. For example, *P. garrettii* is similar to *P. siamensis* due to having a stem without interpetiolar ridges, densely villose hairs on the abaxial surface of the leaves, a glabrous ovary, and clavoid fruits [8]. We found that the fruit length of *P. garrettii* is longer than that of *P. siamensis* (2.89–3.38 mm vs. 2.04–2.63 mm) (Figure 3j and Figure 7d). The presence of an endocarp protrusion distinguishes selected species (types I–III). The shape of sculptured cells on the endocarp surface is also distinct among species having narrowly and broadly obovoid fruits (fruit type I). Uniformly polygonal sculptured cells are observed exclusively in *P. annulata*, *P. cordifolia*, *P. fulva*, *P. mollissima*, *P. odorata*, *P. pubescens*, and *P. serrata*. In contrast, the remaining species exhibit a mixture of sculptured cell shapes and irregular tetragonal and polygonal types.

The species circumscription of *P. serratifolia* has been a subject of debate, considering as the *P. serratifolia* species complex. Several species found in Thailand have been reduced under *P. serratifolia* as synonyms in the previous study, such as *P. cordifolia*, *P. paniculata*, and *P. punctulata* [9]. However, studies such as Leeratiwong et al. [8], Li et al. [3], and Hai et al. [7] recognized these synonyms as distinct species. Our investigations of fruit and endocarp characteristics, as well as evidence from phenetic analyses, support the reinstatement of these species from *P. serratifolia* (Table 1, Figure 1). *Premna cordifolia* has larger fruits than those of *P. serratifolia* (5.32–6.65 mm long and 4.25–5.66 mm wide vs. 3.33–4.89 mm long, 2.58–2.99 mm wide, respectively) (Table 1). The type of endocarp protrusion can also help differentiate between these two species: protrusion type II in *P. cordifolia* (Figure 3d) and protrusion type I in *P. serratifolia* (Figure 7a). Although the width of fruits clearly supports the separation of *P. punctulata* (3.79–5.69 mm wide) from *P. serratifolia* (2.58–2.99 mm wide) (Table 1), the phenetic analyses show the overlapping distribution of these species due to the similarities in the qualitative characteristics (Figure 1). The distinction between *P. paniculata* and *P. serratifolia* is notably supported by the differences in endocarp protrusion (protrusion type II in *P. paniculata* vs. protrusion type I in *P. serratifolia*) (Figure 5d and Figure 7a). The distinction between these ambiguous species can be further supported through morphological investigations, such as examining the presence of interpetiolar ridges on the stems and subsessile glands on the abaxial leaf surface and the number of calyx lobes, as noted by Leeratiwong et al. [8,69]. Further study based on molecular evidence is also required to confirm the taxonomic position of these species within the *P. serratifolia* complex, as well as to explore fruit characteristics of the genus *Premna* in relation to the phylogenetic relationships.

## 4. Materials and Methods

### 4.1. Sample Collection and Herbarium Specimen Preparation

Mature fruits sampled from 21 species of *Premna* in Thailand were collected from fresh field collections and dried herbarium specimens in the following herbaria: the Bangkok Herbarium (BK), Forest Herbarium (BKF), Royal Botanic Gardens, Kew (K), and Herbarium of Prince of Songkla University Herbarium (PSU) (herbarium acronyms according to Thiers [70]). The taxon sampling was based on the synopsis of Thailand by Leeratiwong et al. [8], incorporating updated name changes from subsequent studies [9,10,69,71] (Table 1). Of these, we retained *P. cordifolia*, *P. paniculata*, and *P. punctulata*, which were synonymized under *P. serratifolia* by de Kok [9], as distinct species due to macromorphological and ecological differences. A minimum of 20 fruit specimens were collected for each taxon. However, the number of available specimens was limited for certain taxa: *P. punctulata* (9 specimens), *P. repens* (6 specimens), and *P. siamensis* (10 specimens).

### 4.2. Assessment of Fruit Morphological Characters

Fruit specimens of each species were examined for morphological characteristics, adapted from Krawczyk and Głowacka [14] and Jeon et al. [21]. The number of fruits examined for each taxon is shown in Table 1 (n), although the sampling was limited by the availability of fruits in the selected specimens. The length (L) and width (W) of each fruit were measured using a Vernier caliper. The values of the average length and width, including a standard deviation, were calculated by the functions MAX, MIN, AVERAGE, and STDEV in Microsoft Excel 2016, respectively. The types of fruit based on the development of the mericarp were recorded. After thoroughly examining the external fruit morphology, they were processed to study the endocarp morphology and micromorphology.

### 4.3. Examination of Endocarp Morphology and Micromorphology

The sampled fruits used for studying the endocarp characteristics were soaked in water for at least 10 min to remove the mesocarp and exocarp, the fleshy and soft pericarp layers of *Premna* fruits. The fusion layer of the exocarp and mesocarp was peeled using a needle and forceps and gently exfoliated using a nylon brush (adapted from Yang and Chen [72]). The endocarps were cleaned in NaOCl for 5 min to sanitize the surfaces, then thoroughly rinsed with water. The cleaned endocarps were dried at 60 °C for 3 to 6 h until fully dry. The endocarp morphology was examined under LM (stereomicroscope, Olympus SZ51, Tokyo, Japan) and photographed (EPview version 1.4) to examine the overview and the protrusion types. To study the micromorphology, the endocarps were mounted onto a stub using double-sided adhesive tape, following the methods of Xiang et al. [73], Jeon et al. [21], and Bai et al. [74]. The representatives of each fruit type were cross-sectioned and mounted on the stub to observe the construction within the endocarp layer. The specimens were then sputter-coated with gold using a Cressington 108 Auto Sputter Coater (Cressington Scientific Instruments Ltd., Watford, UK). The micromorphological details of the endocarps, including sculptured cell characteristics, tissue organization, and the presence of pits on cell walls, were analyzed using scanning electron microscopy (SEM), Zeiss Merlin Compact Scanning Electron Microscope (Carl Zeiss AG, Jena, Germany) at an acceleration voltage of 5 kV. The terminology of fruit morphology and micromorphology followed Barthlott [75], Salmaki et al. [76], Krawczyk and Głowacka [14], Beentje [77], and Crang et al. [78].

### 4.4. Statistical Analyses

Qualitative and quantitative data on the fruit and endocarp characteristics obtained 423 accessions were analyzed using R software version 4.3.1 [79] using a factor analysis of mixed data (FAMD), a principal component method designed to explore datasets containing both continuous and categorical variables, facilitating the investigation of similarities among samples [80]. The analyses were performed with the FactoMineR version 2.11 [81], Factoextra version 1.0.7 [82], ggpubr version 0.6.0 [83], and ggplot2 version 3.4.4 packages [84]. The character states incorporated in the statistical analyses are length (mm), width (mm), length-to-width ratio, fruit shape (C = clavoid; BO = broadly obovoid; NO = narrowly obovoid), shape of the sculptured cells on the endocarp (ITP = irregular tetragonal and polygonal; OITOP = obscurely irregular tetragonal and obscurely polygonal; OP = obscurely polygonal; P = polygonal), and protrusion type of the endocarp (I, II, III).

## 5. Conclusions

In this study, investigations into endocarp morphology and micromorphology of 21 species of *Premna* in Thailand, using LM and SEM, along with observations of mature fruit morphology, reveal new taxonomic features for species identification and classification. Phenetic analyses support that fruit shape, shape of sculptured cells on the endocarp, and protrusion type of the endocarp are significant variables for distinguishing certain species. We hypothesize that species having clavoid fruits (fruit type II) may be closely related. The different types of endocarps also refer to alternative seed dispersal modes. The results of this work also support the species delimitation within the *P. serratifolia* complex presented in the previous studies, including *P. cordifolia*, *P. paniculata*, and *P. punctulata*. Clarifying the phylogenetic relationships requires further molecular studies and additional fruit samples from throughout the geographic range of *Premna* distribution to support a taxonomic revision on a monographic scale.

## Figures and Tables

**Table 1:** Detailed investigation of mature fruits and endocarp characters of *Premna* in Thailand.

Species	Shape	Length (mm) Min–Max (Average ± SD)	Width (mm) Min–Max (Average ± SD)	Length/Width Min–Max (Average ± SD)	Sculptured Cell Shape of Endocarp	Protrusion Type	Voucher Specimens
**Fruit type I**							
1. *Premna annulata* H.R.Fletcher (n = 20, Figure 3a–c)	narrowly obovoid	4.23–5.40 (4.83 ± 0.32)	3.24–4.39 (3.66 ± 0.32)	1.09–1.56 (1.33 ± 0.13)	P	I	*Leeratiwong 16-841* (PSU) *Smitinand 2874* (BKF, K) *Maxwell 84-236* (BKF)
2. *Premna cordifolia* Roxb. (n = 20, Figure 3d–f)	narrowly obovoid	5.32–6.65 (5.92 ± 0.42)	4.25–5.66 (4.91 ± 0.40)	1.08–1.40 (1.21 ± 0.08)	P	II	*Leeratiwong 18-1484* (PSU)
3. *Premna fulva* Craib (n = 20, Figure 3g–i)	broadly obovoid	4.29–5.38 (5.04 ± 0.31)	4.04–5.49 (4.76 ± 0.42)	0.92–1.20 (1.06 ± 0.08)	P	III	*Geesink 6843* (K) *Leeratiwong 08-356* (PSU)
4. *Premna herbacea* Roxb. (n = 20, Figure 4a–c)	narrowly obovoid	3.20–4.19 (3.71 ± 0.27)	2.46–3.49 (2.95 ± 0.31)	1.01–1.46 (1.27 ± 0.13)	IT, P	I	*Geesink et al*. *7062* (K) *Kostermans 281* (K) *Leeratiwong 05-240* (PSU) *Suddee et al*. *871* (K)
5. *Premna mollissima* Roth (n = 20, Figure 4g–i)	narrowly obovoid	4.18–5.05 (4.68 ± 0.23)	3.28–4.97 (3.99 ± 0.51)	0.94–1.42 (1.19 ± 0.14)	P	I	*Kostermans 93* (K) *Leeratiwong 05-222* (PSU)
6. *Premna nana* Collett & Hemsl. (n = 20, Figure 4j–l)	narrowly obovoid	3.11–3.93 (3.55 ± 0.23)	2.60–3.77 (3.03 ± 0.32)	0.98–1.42 (1.18 ± 0.11)	IT, P	I	*Leeratiwong 04-2* (KKU) *Leeratiwong 05-217* (PSU, KKU) *Puudjaa et al*. *1908* (BKF)
7. *Premna odorata* Blanco (n = 21, Figure 5a–c)	broadly obovoid	4.94–7.48 (5.79 ± 0.72)	4.65–7.71 (5.90 ± 0.72)	0.88–1.17 (0.98 ± 0.07)	P	II	*Geesink & Santisuk 5434* (K) *Kerr 15383* (K) *Leeratiwong 05-228* (PSU) *Leeratiwong 17-1466* (PSU)
8. *Premna paniculata* H.R.Fletcher (n = 20, Figure 5d–f)	narrowly obovoid	3.22–4.20 (3.70 ± 0.27)	2.33–3.82 (3.23 ± 0.48)	0.98–1.57 (1.17 ± 0.19)	IT, P	II	*Kerr 20536* (K) *Leeratiwong 16-545* (PSU)
9. *Premna pubescens* Blume (n = 34, Figure 5g–i)	broadly obovoid	3.40–5.80 (4.53 ± 0.66)	3.27–6.17 (4.57 ± 0.73)	0.88–1.12 (1.00 ± 0.06)	P	I	*Leeratiwong 05-229* (BKF) *Leeratiwong 05-233* (PSU) *Phonsena et al*. *5926 (BKF)* *Pooma et al*. *1814 (K)* *Pooma et al*. *7077* (K) *Winit 1405* (BKF)
10. *Premna punctulata* C.B.Clarke (n = 9, Figure 5j–l)	narrowly obovoid	4.37–5.70 (5.09 ± 0.42)	3.79–5.69 (4.49 ± 0.68)	0.94–1.30 (1.15 ± 0.11)	IT, P	I	*Leeratiwong 18-1487* (PSU) *Leeratiwong 22-2202* (PSU)
11. *Premna rabakensis* Moldenke (n = 27, Figure 6a–c)	broadly obovoid	3.08–4.75 (3.93 ± 0.57)	2.96–4.25 (3.55 ± 0.39)	0.95–1.28 (1.10 ± 0.08)	IT, P	I	*Leeratiwong 04-16* (PSU) *Geesing & Phengkhlai 6151* (K) *Put 4011* (K)
12. *Premna repens* H.R.Fletcher (n = 6, Figure 6d–f)	narrowly obovoid	4.12–5.83 (5.02 ± 0.73)	3.45–4.68 (4.17 ± 0.54)	1.01–1.31 (1.21 ± 0.12)	IT, P	I	*Leeratiwong 04-46* (PSU) *Leeratiwong 04-155* (PSU
13. *Premna serrata* H.R.Fletcher (n = 20, Figure 6j–l)	narrowly obovoid	2.38–4.03 (3.39 ± 0.44)	2.27–3.26 (2.70 ± 0.28)	1.00–1.48 (1.26 ± 0.12)	OP	I	*Leeratiwong 05-243* (PSU) *Pooma et al*. *5345* (K) *Suddee & Puudjaa 1103* (K) *Tagawa et al*. *T-9920* (K)
14. *Premna serratifolia* L. (n = 22, Figure 7a–c)	narrowly obovoid	3.33–4.89 (4.22 ± 0.35)	2.58–2.99 (2.84 ± 0.11)	1.12–1.75 (1.49 ± 0.12)	IT, P	I	*Satthaphorn 235* (PSU)
15. *Premna stenobotrys* Merr. (n = 20, Figure 7g–i)	narrowly obovoid	4.58–5.62 (5.05 ± 0.35)	3.31–5.20 (4.03 ± 0.49)	1.06–1.40 (1.26 ± 0.10)	IT, P	I	*Larsen & Larsen 33855* (K) *Leeratiwong 05-269* (KKU) *Nielsen et al*. *1926* (BKF, K) *Phonsena et al*. *4661* (BKF)
16. *Premna tomentosa* Willd. (n = 25, Figure 7j–l)	narrowly obovoid	4.02–4.92 (4.51 ± 0.25)	3.14–4.85 (3.97 ± 0.43)	0.97–1.32 (1.14 ± 0.10)	IT, P	I	*Kerr 13117* (K) *Leeratiwong 04-54* (PSU) *Parnell et al*. *95-296* (K) *Prapat 64* (K) *Winit 414* (K)
**Fruit type II**							
17. *Premna garrettii* H.R.Fletcher (n = 20, Figure 3j–l)	clavoid	2.89–3.38 (3.15 ± 0.17)	1.21–1.77 (1.44 ± 0.14)	1.82–2.54 (2.21 ± 0.20)	OIT, OP	III	*Leeratiwong 05-242* (KKU, PSU)
18. *Premna interrupta* Wall. ex Schauer (n = 20, Figure 4d–f)	clavoid	1.52–2.49 (2.11 ± 0.27)	0.98–1.37 (1.13 ± 0.12)	1.48–2.31 (1.88 ± 0.27)	IT, P	III	*Kerr 5379* (K)
19. *Premna scandens* Roxb. (n = 30, Figure 6g–i)	clavoid	3.37–4.47 (4.04 ± 0.31)	1.03–1.87 (1.40 ± 0.22)	1.90–3.81 (2.94 ± 0.48)	OIT, OP	III	*Put 79* (K) *Chantaranothai 329* (KKU)
20. *Premna siamensis* H.R.Fletcher (n = 10, Figure 7d–f)	clavoid	2.04–2.63 (2.42 ± 0.21)	1.12–1.34 (1.23 ± 0.07)	1.63–2.20 (1.98 ± 0.20)	IT, P	III	*Sutheesorn 2441* (BK)
21. *Premna trichostoma* Miq. (n = 20, Figure 8a–c)	clavoid	2.99–3.95 (3.39 ± 0.26)	1.32–1.86 (1.52 ± 0.14)	1.90–2.60 (2.25 ± 0.24)	IT, P	III	*Leeratiwong 05-237* (PSU) *Leeratiwong 06-335* (PSU) *Vidal & Niyomdham 6351* (K)

Abbreviations: IT = irregular tetragonal, OIT = obscurely irregular tetragonal, OP = obscurely polygonal, P = polygonal.

## Data Availability

All data in this study is included in this article.

## Supplementary Materials

The following supporting information can be downloaded at https://www.mdpi.com/article/10.3390/plants14111706/s1: Figure S1: FAMD results showing the contributions of the six fruit and endocarp characters of fruits and endocarps towards the first and second dimensions.
