# Supplementary material for: Endocarp Morphology of Premna (Lamiaceae) in Thailand and Its Taxonomic Significance
Source: Plants (Basel). 2025 Jun 3;14(11):1706. doi: 10.3390/plants14111706 (PMC12158171; doi:10.3390/plants14111706)
Supplement: Supplementary file 1 [file plants-14-01706-s001.zip › plants-3643720-supplementary.pdf]

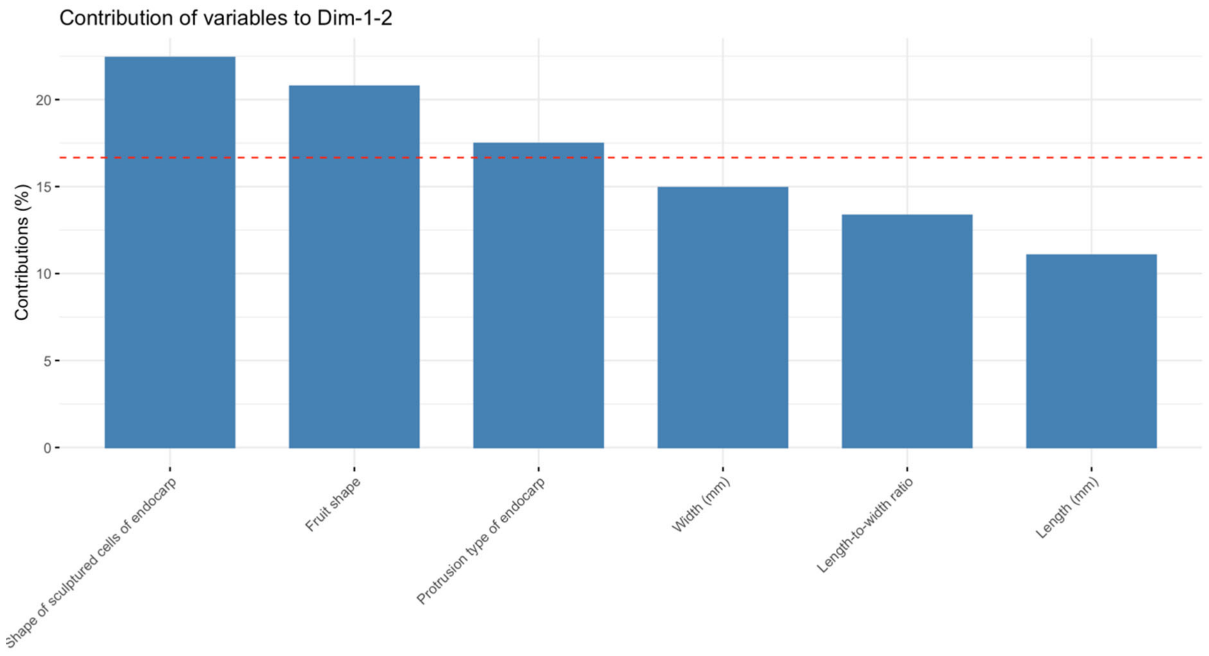

Figure S1. FAMD results showing the contributions of the six fruit and endocarp characters of fruits and endocarps towards the first and second dimensions. The dashed lines represent the threshold value generated by the algorithm.
